# Randomized, Double-Blind, Placebo-Controlled Parallel Clinical Trial Assessing the Effect of Fructooligosaccharides in Infants with Constipation

**DOI:** 10.3390/nu10111602

**Published:** 2018-11-01

**Authors:** Daniela da Silva Souza, Soraia Tahan, Thabata Koester Weber, Humberto Bezerra de Araujo-Filho, Mauro Batista de Morais

**Affiliations:** 1 Division of Pediatric Gastroenterology, Escola Paulista de Medicina, Universidade Federal de São Paulo, São Paulo 04023-062, Brazil; danielassouza@gmail.com (D.d.S.S.); s.tahan@uol.com.br (S.T.); biogene@gmail.com (H.B.d.A.-F.); 2 Instituto de Biociências, Universidade Estadual Paulista, Botucatu, São Paulo 18618-689, Brazil; thabataweber@yahoo.com.br

**Keywords:** constipation, prebiotic, intestinal transit time, infant, *Bifidobacterium*

## Abstract

Constipation often begins in the first year of life. The aim of this study was to assess the effect of fructooligosaccharides (FOS) in the treatment of infants with constipation. This randomized, double-blind, placebo-controlled clinical trial included infants with constipation who were randomly assigned to one of two parallel groups: FOS or placebo. Either the FOS supplement or the placebo was added to the infant formula. Thirty-six infants completed the 4-week intervention. Therapeutic success occurred in 83.3% of the FOS group infants and in 55.6% of the control group infants (*p* = 0.073; one-tailed test). Compared with the control group, the FOS group exhibited a higher frequency of softer stools (*p* = 0.035) and fewer episodes of straining and/or difficulty passing stools (*p* = 0.041). At the end of the intervention, the mouth-to-anus transit time was shorter (22.4 and 24.5 h, *p* = 0.035), and the Bifidobacterium sp. count was higher (*p* = 0.006) in the FOS group. In conclusion, the use of FOS in infants with constipation was associated with significant improvement in symptoms, but the results showed no statistical significance regarding the success of the therapy compared with the control group. FOS was associated with reduced bowel transit time and higher counts of the genus Bifidobacterium in the stool.

## 1. Introduction

Constipation occurs frequently in childhood and accounts for approximately 25% of visits to pediatric gastroenterology outpatient clinics [1,2,3]. In most cases, symptoms appear during the first year of life [3,4,5]. This stage of life involves significant changes to the infant’s diet, including the introduction of complementary feeding and inappropriate early interruption of breastfeeding [6].

Breastfeeding protects against the development of constipation and is associated with higher stool frequency and softer stools during the first six months of life [5,7,8]. The lower constipation frequency among breastfed infants might be due to the ingestion of prebiotic oligosaccharides, which are the third largest component of breast milk and are absent from nonhuman milk [9]. Oligosaccharides have a bifidogenic effect (contributing to the growth of beneficial bacteria from the genera *Bifidobacterium* and *Lactobacillus*) and modify intestinal metabolic activity by reducing the pH and increasing the short-chain fatty acid concentration. This modulation of the gut microbiota may contribute to increasing the stool bulk and intestinal motility, thus facilitating the passage of stool [10,11].

Considering that human breast milk contains large amounts of oligosaccharides, a mixture of prebiotic oligosaccharides (galactooligosaccharides and fructooligosaccharides) has been added to some infant formulas. The effect of this mixture on the gut microbiota and bowel pattern was assessed. The results indicated an increased stool frequency [12,13,14,15,16,17] and softer stools [12,13,14,15,16,17] in the infants who were fed the prebiotic mixture. In addition, the number of fecal *Bifidobacteria* was higher in the group fed the prebiotic mixture than in the control group [13,17]. A recent clinical study conducted in Brazil assessed the effect of galactooligosaccharides on constipation among 6 to 14-year-old children and adolescents. The results showed higher stool frequency, less straining and softer stools during the prebiotic intake period [18].

Although several studies have demonstrated the influence of prebiotics on stool frequency and consistency, no randomized, double-blind, placebo-controlled clinical trial has assessed their use in treating infants with constipation. Therefore, this clinical trial assessed the effect of prebiotic FOS on the bowel pattern of infants (6–24 months old) with constipation as well its effects on mouth-to-anus transit time and stool counts of the genera *Bifidobacterium* and *Lactobacillus*.

## 2. Materials and Methods 

### 2.1. Study Design

This study was a randomized, double-blind, placebo-controlled, parallel trial that complied with the guidelines formulated by the Consolidated Standards of Reporting Trials (CONSORT) [19]. The trial was registered at the Brazilian Registry of Clinical Trials (registry number RBR-2x8wqc; registry URL: http://www.ensaiosclinicos.gov.br/rg/RBR-2x8wqc/).

Infants aged 6–24 months were assessed for inclusion in the study at basic health units or daycare centers in the cities of Osasco and São Vicente (State of São Paulo, Brazil). Constipation was defined as the elimination of hard stools associated with one of the following characteristics: pain or straining while passing stools, scybalous stools, cylindrical and cracked or cylindrical and thick stools and stool frequency less than three times per week, as per the modified international recommendations [20] used in previous studies [5,21]. After inclusion in the study and before randomization, a daily register of intestinal habits was tracked in a diary for one week and used to confirm the constipation diagnosis. 

Infants who fulfilled any of the following criteria were not admitted to the study: (1) exclusively or partially breastfeeding; (2) use of antibiotics, dietary fiber or prebiotic supplements in the past 30 days; (3) iron deficiency anemia (hemoglobin < 11.0 g/dL); 4. current use of medications that can cause constipation (except ferrous sulfate); 5. malnutrition or obesity; 6. infants whose parents were not able to record the infant’s bowel pattern during follow-up.

Other exclusion criteria were the clinical need for prescribed laxatives, a diagnosis of fecaloma or FOS or placebo intake below 80%.

### 2.2. Intervention

Randomization was performed by a health professional who did not participate in the study. A computer-generated random number table was used, and the participants were assigned to blocks by body weight (6.0–8.9 kg, 9.0–11.9 kg and over 12.0 kg). In each weight interval, a block of four participants was assigned to the study and control groups at 2:2 ratios. The prebiotic and placebo were delivered in identical packaging, and the coding was standardized according to the random number table. The participants received the corresponding intervention based on their order of entry into the study. Neither the participants nor the investigators were aware of the intervention (prebiotic or placebo). 

The study lasted five weeks with one week for clinical evaluation before randomization. During the intervention, participants received a prebiotic composed of 100% FOS (the FOS polymerization degree ranged from two to six monosaccharide molecules) or a placebo composed of 100% (flavorless) maltodextrin for four weeks. The FOS and placebo doses were 6, 9 or 12 g daily based on the infants’ weight groups of 6.0–8.9 kg, 9.0–11.9 kg or over 12.0 kg, respectively. Both were supplied by the same manufacturer (FQF, Farmoquimica, São Paulo, Brazil). Based on the randomization, each patient received the FOS or placebo in two doses. The FOS or placebo supplements were administered in baby bottles and dispersed in infant formula (Milupa^R^, Danone, Brazil) or cow’s milk (Ninhofortificado^R^, Nestlé, Brazil) for infants aged less than 12 months and older than 12 months, respectively.

### 2.3. Study Stages

The study involved three stages: baseline, intervention and the last week of the clinical trial. At baseline (first week), clinical interviews were conducted. The interviews included characterization of the infants’ bowel patterns and 24-h diet recall; stool samples were also collected, and hemoglobin levels in capillary blood samples and the carmine dye mouth-to-anus transit time were measured. The infants’ parents received home monitoring diaries. During the baseline week, the participants received infant formula or iron-fortified cow’s milk without supplemental FOS or the placebo. This procedure permitted evaluation of the effect of dietary changes on the infants’ bowel patterns. Only infants who continued to experience constipation at the end of the first week were included in the intervention. 

The intervention period was conducted over the following four weeks and included one weekly clinical evaluation. Participants received FOS or the placebo as indicated by the randomization. During the intervention, the infants’ guardians recorded the bowel patterns and adverse effects in their home monitoring diaries. 

During the end-of-study period (the last week of the intervention period), a second stool sample was collected, the carmine dye mouth-to-anus transit time was measured, and 24-h diet recall was recorded. 

### 2.4. Study Procedures

The infants’ bowel patterns were assessed based on data provided by their guardians at clinical interviews based on their home monitoring diaries.

Hemoglobin concentration in the capillary blood samples was measured using a photometer (HemoCue, Angelholm, Sweden). Infants with anemia (hemoglobin concentration less than 11 g/dL per the World Health Organization [21]) were excluded. Anemia was evaluated and treated by pediatricians at basic health units. The guardians of infants without anemia who were receiving ferrous sulfate at prophylactic doses were directed to discontinue the medication during the study period. 

The mouth-to-anus transit time, expressed in hours, was measured using carmine dye (Certistain, Merck, Brazil). The dye was administered at a dose of 0.25 g dissolved in 50 mL of water [22]. Guardians were directed to record the dates and times at which the infants ingested the dye and first expelled red-stained stools. 

The infants’ typical diets were assessed using the 24-h recall method [23]. Energy, macronutrient (carbohydrate, fat and protein) and micronutrient (calcium and iron) intake estimates were calculated using Nutrition Decision Making Support System software (version 2.5) (Universidade Federal de São Paulo, São Paulo, Brazil). Dietary fiber intake was calculated using the Brazilian Food Composition Table (Tabela Brasileira de Composição de Alimentos) [24], which lists the dietary fiber content of foods according to the Association of Official Analytical Chemists’ (AOAC) enzymatic-gravimetric method [25].

Body weight and height were measured at all clinical evaluations. Infants were weighed without clothes using digital scales (Filizola, São Paulo, Brazil) with 15-kg capacities and 5-g sensitivities. Body length was measured using a portable, horizontal 100-cm stadiometer with 0.1-cm precision. The weights and lengths of the infants are expressed as weight-per-age, length-per-age and weight-per-length z-scores. Z-scores were calculated using the World Health Organization’s Anthro software, version 3.2.2 (Geneva, Switzerland) [26].

The guardians who received instructions on delivering the FOS or placebo were also asked to record the infants’ bowel patterns (stool frequency and consistency, pain, crying, straining or difficulty passing stools) and adverse effects (excessive crying, regurgitation or vomiting) each day in their home monitoring diaries. To facilitate description of stool shape and consistency, a scale with four figures illustrating infant stools was appended to the home monitoring diary.

For determining the number of *Bifidobacterium* and *Lactobacillus*, stool samples were collected by the guardians according to established guidelines. Approximately 1 g of each stool sample was transferred to a microtube containing ASL buffer from the DNA extraction QIAamp Mini Stool Kit, and the sample was stored at −20 °C until the DNA was to be extracted. The bacterial genomic DNA was extracted according to the protocol recommended by the extraction kit manufacturer (Qiagen, Hilden, Germany), and the purified DNA was diluted in a buffer solution to a final volume of 200 µL. The DNA concentration was measured using a NanoDrop 1000 spectrophotometer (Thermo Scientific, Waltham, MA, USA). All DNA samples were diluted to a final concentration of 20 ng/µL and stored at −20 °C.

DNA from all fecal samples was subjected to real-time polymerase chain reaction (qPCR). The primers used were selected to identify and quantify *Lactobacillus* spp. [27] and *Bifidobacterium* spp. [28]. All reactions were performed in duplicate in a final volume of 10 µL that included 5 µL of Rotor-gene SYBR Green PCR Master Mix (Qiagen, Hilden, Germany). Thermocycling was performed in a Rotor-gene Q device (Qiagen, Hilden, Germany) with the following parameters: 5 min at 95 °C and 40 cycles of 95 °C for 10 s and 60 °C for 15 s. The dissociation protocol used to obtain the melting curve was 95 °C for 1 min followed by variation of the temperature from 70 °C to 95 °C with a temperature increase of 1 °C/s. The negative control contained all the ingredients except the DNA sample. The standard curve for all of the analyses was created by amplifying a Topo TA plasmid (Invitrogen, ThermoFisher, Waltham, MA, USA) carrying a fragment of the reference gene previously amplified by conventional PCR, and its specificity was confirmed by sequencing and BLAST system alignment. With the molecular mass of the plasmid and insert known, it is possible to calculate the copy number as follows: mass in daltons (g/mol) = (size of double-stranded (ds) product in base pairs (bp)) (330 Da × 2 nucleotides (nt)/bp) [29,30,31]. If the copy number and the concentration of the plasmid DNA are known, the number of molecules added to subsequent real-time PCR runs can be calculated, thus providing a standard for determining the copy numbers of specific genes [29,30,31]. The real-time PCR results are expressed as log CFU per gram of stool (log CFU/g) using the average number of copies of 16S rRNA genes in each bacterium to normalize the counts [29,30,31].

### 2.5. Outcomes

The primary outcome was therapeutic success defined as a normal bowel pattern at the end of the study, i.e., predominantly soft, amorphous or cylindrical stools without cracks as well as the absence of pain or difficulty passing stools (a pattern incompatible with the criteria adopted herein for characterizing constipation). Therapeutic failure was defined as the persistence of a bowel pattern indicating constipation at the end of the study or a clinical need for laxatives during the intervention period.

Secondary outcomes were stool frequency, stool consistency, pain and/or crying when passing stools, and difficulty and/or straining while passing stools during the last week of the clinical trial. The mouth-to-anus transit time and *Bifidobacterium* spp. and *Lactobacillus* spp. counts were measured before and at the end of the intervention.

### 2.6. Ethical Issues

The study protocol was approved by the Research Ethics Committee of the Universidade Federal de São Paulo (Federal University of São Paulo). Written informed consent was obtained from the parents of the infants prior to inclusion in the study. 

### 2.7. Statistical Analysis

Sample size was calculated based on the primary outcome (therapeutic success). A success rate of 70% was assumed for the group that received FOS, and a success rate of 20% was assumed for the control group. For α = 0.05 and β = 0.20 (power = 0.80), each group should include 19 individuals. 

To compare the mean and median values between groups, parametric (Student’s *t*-test) or non-parametric (Mann–Whitney U) tests were used based on the data distribution. A chi-squared test was used to compare proportions. For the variables therapeutic success, stool frequency, stool consistency, and occurrence of pain and/or crying when passing stools or difficulty and/or straining when passing stools, the study goal was to establish whether the FOS performed better than the placebo. Therefore, p-values from one-tailed tests were used to compare those variables. Calculations were performed using SigmaStat 3.1 (Systat, San Jose, CA, USA) and EpiInfo (CDC, Atlanta, GA, USA). Differences between groups were considered significant when *p* < 0.05. 

## 3. Results

### 3.1. Patients

**Figure 1:**
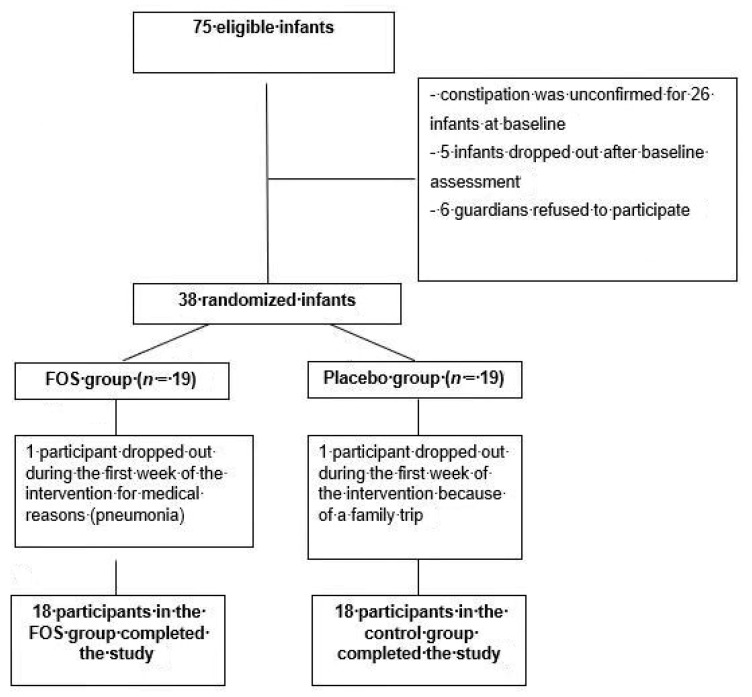
Flowchart showing the study participants. FOS: fructooligosaccharide.

Seventy-five infants were eligible for inclusion in the study; however, the constipation diagnosis was unconfirmed in 26 (34.7%) cases during the first week’s assessment, five guardians dropped out during the baseline period, and six did not consent to participate. Thus, 38 infants were randomly assigned to the two groups. The study group (*n* = 19) received FOS, and the control group (*n* = 19) received the placebo. No statistically significant differences were found between the groups at the time of inclusion in the study (after the end of the baseline period) (Table 1). During the intervention period, one participant in the FOS group dropped out for medical reasons (pneumonia), and one participant in the control group dropped out due to a family trip. Therefore, 36 infants completed the clinical trial: 18 in the FOS group and 18 in the control group (Figure 1).

### 3.2. Primary Outcome

Therapeutic success occurred more frequently in the FOS group (15/18, 83.3%) than in the control group (10/18, 55.6%); however, this difference was not statistically significant (*p* = 0.073, one-tailed chi-squared test).

### 3.3. Secondary Outcome

For the secondary outcomes, all variables were similar at admission (Table 2). The weekly stool frequencies in the last week of the study were similar in both groups, and no statistically significant increment in the number of bowel movements occurred between the baseline and the last week of the study. Both groups presented fewer (*p* < 0.05) bowel movements with pain/crying or straining/difficulty when passing stool and increments (*p* < 0.05) in the percentages of bowel movements with soft stool. At the last week of the study, the FOS group presented fewer bowel movements with straining/difficulty (*p* = 0.041) and a higher percentage of passing soft stools than the control group (*p* = 0.035).

The median mouth-to-anus transit times for the FOS and control groups were similar (*p* = 0.740, two-tailed Mann-Whitney test) upon admission in the study, at 23.3 h (percentiles 25 and 75: 22.2; 25.6) and 23.5 h (percentiles 25 and 75: 21.9; 27.0), respectively. At the end of the study, the mouth-to-anus transit time was lower (*p* = 0.035; one-tailed Mann–Whitney test) in the FOS group (median: 22.4 h; percentiles 25 and 75: 18.3; 25.7) than in the control group (median: 24.5 h; percentiles 25 and 75: 23.0; 33.3).

At the end of the study, the number of bacteria of the genus *Bifidobacterium* was higher in the FOS group than in the control group (Table 3).

The z-scores (weight-for-height, weight-for-age, height-for-age and body mass index-for-age) and the food intake as per the 24-h food recall method were similar in the FOS and control groups at baseline and at the end of the study (data not shown). Dietary fiber intake (excluding the FOS supplement used in the intervention) was 6.6 ± 2.1 g/day and 7.4 ± 2.4 g/day at admission (*p* = 0.269) in the FOS and control groups, respectively. At the end of the study, these values were 6.5 ± 2.3 g/day and 7.1 ± 2.2 g/day (*p* = 0.457), respectively.

### 3.4. Adverse Effects

All participants who completed the 4-week intervention (*n* = 36) consumed more than 80% of the delivered amount of FOS or placebo. Adverse effects were reported only in the FOS group (two infants with abdominal distension and flatulence), but the treatment was continued. One of the infants in the FOS group had two vomiting episodes during the first week of intervention.

## 4. Discussion

This clinical trial showed that FOS intake contributed to relieving infant constipation (increased frequency of softer stools) and reducing the number of defecation events with straining and/or difficulty passing stools. FOS also reduced the bowel transit time and increased the number of *Bifidobacterium* in constipated infants who were treated with FOS relative to the control group. The stool frequencies in the two groups were similar at baseline and at the last week of the study. Although the difference did not reach statistical significance, therapeutic success was more frequently achieved in the group treated with FOS than in the control group. 

Per the 2014 ESPGHAN/NASPGHAN guideline for constipation, routine use of prebiotics is not recommended for treating childhood constipation. Recently, a clinical trial performed in Brazil [17] showed a positive effect of galactooligosaccharide probiotics on constipation in children and adolescents aged 4–16 years. That study showed softer stools, increased stool frequency and less pain or difficulty when passing stools during the prebiotic intake period. The present study showed similar results except for stool frequency. However, the defecation frequency of constipated infants included in our study was not reduced; therefore, no increase in the bowel movement frequency was expected.

Prebiotics were administered to healthy bottle-fed infants in previous clinical trials [12,13,14,15,16] to assess their influence on bowel patterns. Infants who received prebiotic mixtures of galacto- and fructooligosaccharides exhibited softer stools. Our results agreed with these studies, as the frequency of softer stools increased after four weeks of intervention only with FOS. Additionally, the mixture used in the infant formula contained 90% galactooligosaccharides and only 10% FOS. 

The increased frequency of softer stools and the consequent reduction in defecation events with straining and/or difficulty when passing stools in the FOS-treated group might be related to the effect of this prebiotic on the gut microbiota. Prebiotics are carbohydrates that are not digested in the gastrointestinal tract and are used as an energy source by the gut bacteria [10]. Other studies found that consumption of a mixture of galacto- and fructooligosaccharides increased the number of *Bifidobacterium* [12,13]. Our results showed that at the end of the study *Bifidobacterium* numbers were higher in the FOS group. Thus, only FOS had a prebiotic effect as observed for the galacto- and fructooligosaccharide mixture. This effect might be associated with the short-chain fatty acids produced by the fermentation of prebiotics. The modulation of gut microbiota may contribute to increasing the stool bulk and may stimulate intestinal motility, thus facilitating stool expulsion [10,32].

The daily FOS dose varied between 6 g and 12 g according to the infants’ weights. Infant formula containing 8 g/L of the galacto- and fructooligosaccharide mixture provides 6 g of prebiotic in 750 mL. Therefore, the dose of FOS used in the present study was similar to or higher than the amount that would be provided by regular daily intake of 750 mL of infant formula containing 8 g/L of the galacto- and fructooligosaccharide mixture. The supplemented FOS was well tolerated since only one infant presented two vomiting episodes during the first week of intervention. Mild flatulence and abdominal distension were observed in two FOS group patients; however, the treatment was continued. Therefore, the FOS supplementation was well tolerated, with mild adverse effects in few infants.

The limitations of the present study include the following. The sample may have been insufficient to demonstrate statistical significance for therapeutic success in the FOS and control groups. The therapeutic success observed in the FOS group (83.3%) was similar to the expected value (80%) used to estimate the sample size. The number of infants included in the trial complied with the calculated sample size; however, the proportion of constipated infants in the control group who had therapeutic success (55.6%) was much higher than the value (20%) used in the sample size estimate. The guidance for healthy eating for infants provided at the time of inclusion in the study and the substitution of cow’s milk for infant formula in patients younger than 12 months likely had an effect on improving the constipation. This might also explain the high number of infants (26/75; 34.7%; Figure 1) who were not confirmed to have constipation after the first week of clinical observation (prior to randomization). The macronutrient intake, including dietary fiber, assessed by the 24-h food recall method was similar in the FOS and control groups before and at the end of the intervention. The dietary fiber intake (soluble and insoluble) varied between 6.5 and 7.4 g/day. The suggested daily intake of dietary fiber for this age group is 5–10 g [33]. Notably, neither the American Health Foundation [34] nor the Dietary Recommended Intake [35] recommend specific amounts of dietary fiber intake during the first year of life. Therefore, the quantity of macronutrients, including dietary fiber, consumed by the participants in this study may not explain the decreased constipation. Additionally, the type of milk consumed by all infants was changed at the beginning of the first week, corresponding to the baseline of the clinical trial. Most infants had been fed cow’s milk containing added starch and sugar, which is a frequent feeding habit for infants in Brazil [36,37]. The change in infant formula or the use of fortified cow’s milk in the correct dilution may explain the decreased constipation in the first week and in the control group during the intervention period. These results should not be extrapolated to severely constipated infants since this clinical trial did not include infants requiring other laxatives or fecal disimpaction.

## 5. Conclusions

In conclusion, the use of FOS in infants with constipation was associated with significant improvement in symptoms, but the results showed no statistical significance regarding the success of the therapy compared with the control group. However, the improved stool consistency, the reduced number of episodes of straining and/or difficulty passing stools and the reduced bowel transit time detected among the infants fed FOS are relevant findings that will contribute to preventing more severe constipation symptoms among constipated infants. Additional clinical trials assessing the effect of prebiotics on infant constipation are needed to confirm the results of this study and to provide data for a future meta-analysis.

## Figures and Tables

**Table 1:** Demographic and clinical characteristics at baseline.

	FOS Group (*n* = 19)	Control Group (*n* = 19)	*p*
Age (months)	12.77 ± 4.37	13.00 ± 5.19	0.890 †
Sex			
Female	10 (52.6%)	10 (52.6%)	1.000 *
Male	9 (47.4%)	9 (47.4%)	
Number of bowel movements per week	6.27 ± 1.32	5.66 ± 1.87	0.133 †
Predominant stool shape and consistency			
Cylindrical with cracks	13 (68.4%)	8 (42.1%)	0.191 *
Scybalous	6 (31.6%)	11 (57.9%)	
Straining and/or difficulty in more than 50% of bowel movements	16 (84.2%)	16 (84.2%)	1.000 *
Pain and/or crying in more than 50% of bowel movements	11 (57.9%)	12 (63.2%)	1.000 *

FOS: fructooligosaccharide; Consistency and shape: predominant occurrence (more than four times per week); * Two-tailed chi-squared test with Yates’ correction; † Two-tailed Student’s *t*-test, mean ± standard deviation.

**Table 2:** Clinical secondary outcomes at baseline and at the end of the study (stool frequency, percentage of straining, difficulty, pain, crying during defecation and percentage of soft stools).

	FOS Group (*n* = 18)	Control Group (*n* = 18)	*p* *
Number of bowel movements per week			
Baseline week	6.27 ± 1.32	5.66 ± 1.87	0.133
Last week of the study	6.33 ± 1.28	6.11 ± 1.53	0.320
Pain/crying when passing stools (percent of bowel movements)			
Baseline week	55.13 ± 44.07	60.09 ± 44.44	0.369
Last week of the study	14.68 ± 29.15	28.39 ± 43.82	0.138
Straining/difficulty when passing stools (percent of bowel movements)			
Baseline week	84.47 ± 29.39	79.47 ± 37.63	0.330
Last week of the study	29.65 ± 41.73	55.07 ± 43.44	0.041
Soft stool consistency (percent of bowel movements)			
Baseline week	12.12 ± 15.91	16.92 ± 15.07	0.180
Last week of the study	73.38 ± 29.38	55.38 ± 36.32	0.035

* The data are presented as mean ± standard deviation (One-tailed Student’s *t*-test).

**Table 3:** Secondary outcome: *Bifidobacterium and Lactobacillus* genus counts (log CFU/g).

	FOS Group (*n* = 18)	Control Group (*n* = 18)	*p*
*Bifidobacterium*			
Baseline	6.39 (5.25–8.36)	6.61 (4.48–7.99)	0.301
End of the study	7.37 (5.86–8.43)	5.60 (4.46–6.42)	0.006
*Lactobacillus*			
Baseline	6.27 (4.33–7.54)	6.03 (2.95–7.23)	0.248
End of the study	6.45 (4.83–7.61)	5.39 (3.37–6.73)	0.095

The data are presented as median values with the 25th and 75th percentile values in parentheses (Mann-Whitney test).
